# Detection of muscular system adverse reaction signals in sacubitril/valsartan treatment combined with statins

**DOI:** 10.3389/fphar.2024.1393616

**Published:** 2024-07-15

**Authors:** Fukun Zhao, Min Luo, Yuanmin Wang, Mu Su, Fei Tang

**Affiliations:** ^1^ Department of Clinical Pharmacy, Third Affiliated Hospital of Zunyi Medical University (The First People’s Hospital of Zunyi), Zunyi, Guizhou, China; ^2^ Department of Pain Medicine, Suiyang County Hospital of Traditional Chinese Medicine, Zunyi, Guizhou, China

**Keywords:** sacubitril/valsartan, statins, drug-drug interactions, adverse drug reaction, muscular system

## Abstract

**Objective:**

To detect muscular system adverse reaction signals of sacubitril/valsartan treatment combined with statins (atorvastatin, rosuvastatin, simvastatin) to provide a reference for clinical administration.

**Methods:**

Multiplicative and additive models were used to mine the FDA’s spontaneous reports database to detect signals of drug-drug interactions between sacubitril/valsartan and statins. SAS 9.4 software was used to conduct statistical tests for suspicious signals to determine whether the signals were statistically significant.

**Results:**

A total of 8,883,870 adverse reaction reports were analyzed. The combinations “sacubitril/valsartan - simvastatin - musculoskeletal muscle pain” had statistically significant correlation signals in both models (*P* < 0.05). The combination “sacubitril/valsartan - atorvastatin - myopathy” and “sacubitril/valsartan–simvastatin - myopathy” had statistically significant correlation signal in the multiplicative model (*P* < 0.05).

**Conclusion:**

Compared with a single drug, coadministration of sacubitril/valsartan with atorvastatin may increase safety risks to myopathy, with simvastatin may increase safety risks to the musculoskeletal pain and myopathy, which should be closely monitored in clinical practice.

## 1 Introduction

The combination drug sacubitril/valsartan was the first angiotensin receptor-enkephalinase inhibitor with a dual mechanism of action. Clinical studies have shown that sufficient doses of sacubitril/valsartan have promising therapeutic effects on patients with chronic heart failure (Piepoli et al., 2021; Zheng et al., 2021; Song et al., 2022; Correale et al., 2023). Sacubitril has an inhibitory effect on organic anion transporting polypeptides 1B1 (OATP1B1) and 1B3 (OATP1B3) *in vitro* (Ayalasomayajula et al., 2016). However, many patients with chronic heart failure also take statins (e.g., atorvastatin, rosuvastatin) for secondary prevention of coronary heart disease (Brunetti et al., 2015), and statins are substrates of OATP1B1 and OATP1B3 (Ciută et al., 2023). Therefore, combining sacubitril/valsartan with statins may result in unwanted drug-drug interactions (DDIs), potentially increasing the risk of adverse events (AEs) like harmful reactions in the muscular system. However, there are few studies evaluating the safety of combination treatment with sacubitril/valsartan and statins. Therefore it is necessary to collect further safety data on the combination of these drugs in the real world.

With the rapid development of artificial intelligence, data mining methods have been widely used for early and accurate detection of adverse drug reaction (ADR) signals. However, current ADR data mining mainly focuses on single drugs. In spontaneous reporting systems (SRS), ADRs caused by DDIs account for about 30% of all ADRs, posing a greater risk than single drug use (Srinivasan et al., 2013). Therefore, mining signals of these ADRs is crucial for avoiding large-scale drug injury events caused by combined drug use and ensuring patient safety. The multiplicative and additive models were proposed by Thakrar et al. (2007). They are appropriately extended according to the principle of proportional reporting ratios and applied to the signal detection of adverse drug reactions (ADR) of combined drugs. Such models have good consistency for signal screening, and the results are easy to interpret from a public health perspective, and they were previously validated in FAERS using eight DDIs, four known clinically relevant interactions (e.g., simvastatin–cyclosporine and myopathy) and four drug–event combinations without evidence of interaction (e.g., fexofenadine–ketoconazole and torsade de pointes) (Thakrar et al., 2007). One has been adopted by the WHO to mine combination drug ADR signals (YN, 2021). Currently, there are no reports on using additive and multiplicative models to explore muscle system adverse reactions caused by the combination of sacubitril/valsartan and statins. Accordingly, the present study used data from the US Food and Drug Administration Adverse Event Reporting System (FAERS) database, a rich dataset that can be effectively used for monitoring and evaluating the safety risks of drugs after marketing. Additive and multiplicative models were used to detect the muscular system ADR signals of sacubitril/valsartan combined with statins (atorvastatin, rosuvastatin, or simvastatin) to provide a reference for clinical use.

## 2 Materials and methods

### 2.1 Data sources

Muscular system adverse events resulting from combination treatment with sacubitril/valsartan and statins (atorvastatin, rosuvastatin, or simvastatin) were obtained through the publicly available FAERS database. Adverse events were coded by the Medical Dictionary for Regulatory Activities (version 26.0).

### 2.2 Search strategy

We used OpenVigil 2.1 (http://h2876314.stratoserver.net:8080/OV2/search/) to extract data from the FAERS database. Reports from the marketing time of the drug to the second quarter of 2023 and the generic name of the drug were used for retrieval: sacubitril/valsartan, atorvastatin, rosuvastatin, and simvastatin. Search terms for muscular system adverse reactions included muscle spasms, myalgia, muscular weakness, musculoskeletal pain, rhabdomyolysis, myopathy, blood creatine phosphokinase increased, myositis, and musculoskeletal discomfort.

### 2.3 Research methods

#### 2.3.1 Additive model

The additive model assumes that if the excess risk of drug A is independent of drug B, then there is no DDI. This can be expressed as risk (A, not B) – risk (not A, not B) = risk (A, B) – risk (not A, B). It can be inferred that: risk (A, B) – risk (not A, not B) = [risk (A, not B) – risk (not A, not B)] + [risk (not A, B) – risk (not A, not B)]; that is, RDAB = RDA + RDB. Among them, RDAB = risk (A, B) – risk (not A, not B) represents the excess risk when the two drugs are combined, RDA represents the excess risk when drug A is used alone, and RDB represents the excess risk of drug B alone. RDAB > RDA + RDB suggests a higher risk due to combined drug use. The statistical test of suspicious DDI signals in this model was obtained by constructing the following linear regression model: Risk of event = α + β (drug A) + γ (drug B) + δ (drug A and B) + other covariates, where α is the intercept, also known as the constant term; β, γ, and δ are partial regression coefficients, and other covariates are optional. A measure of drug interaction is the coefficient δ, which indicates that the excess risk of a combination exceeds the sum of the excess risks of two drugs taken alone. When the coefficient δ deviates statistically from 0, especially when δ is greater than 0, it indicates a positive drug interaction.

#### 2.3.2 Multiplicative model

The multiplicative model assumes that if the relative risk of drug A is independent of drug B, then there is no DDI. This can be expressed as risk (A, not B)/risk (not A, not B) = risk (A, B)/risk (not A, B). It can be inferred that: RRAB = RRA × RRB. Among them, RRA = risk (A, not B)/risk (not A, not B) is the relative risk of drug A alone. Assuming no DDI between the drugs A and B, the relative risk of a combination is equal to the product of the relative risks of the two drugs when used alone. Therefore, a statistical difference between RRAB/(RRA × RRB) and 1 indicates a suspicious interaction signal in the drug combination, and a ratio greater than 1 indicates a positive interaction. The statistical test of suspicious DDI signals in this model was obtained by constructing a log-linear regression model: Log (risk of event) = α + β (drug A) + γ (drug B) + δ (drug A and B) + other covariates. If the coefficient δ is statistically significant compared with 0, it indicates that there is an interaction signal. δ > 0 indicates that there is a positive interaction between drug combinations. δ < 0 indicates that the relative risk of the combination drug is lower than the product of the relative risk of the two drugs used alone. The difference between the exponential function exp (δ) and 1 is statistically significant (*P* < 0.05), indicating the existence of an interaction; exp (δ) > 1 indicates the existence of a positive interaction between drugs.

## 3 Results

### 3.1 Search results

A total of 8,883,870 ADR reports were collected from the FAERS database: 68,726 of sacubitril/valsartan, 82,809 of atorvastatin, 77,853 of rosuvastatin, and 1,02,851 of simvastatin. There were 788 ADRs reported with sacubitril/valsartan combined with atorvastatin, 1,022 ADRs reported with sacubitril/valsartan combined with rosuvastatin, and 854 ADRs reported with sacubitril/valsartan combined with simvastatin. The original data are shown in Table 1.

**TABLE 1:** Risk proportions of muscular system adverse events after coadministration of sacubitril/valsartan and statins.

Drug A	Drug B	Adverse reaction	A and B	A, no B	B, no A	No A, no B
sacubitril/valsartan	atorvastatin	muscle spasms	8/788	326/68,726	1715/82,809	78,652/8,883,870
		myalgia	6/788	264/68,726	2,514/82,809	68,379/8,883,870
		muscular weakness	7/788	281/68,726	1,217/82,809	44,118/8,883,870
		musculoskeletal pain	3/788	119/68,726	554/82,809	24,744/8,883,870
		rhabdomyolysis	5/788	62/68,726	550/82,809	13,921/8,883,870
		myopathy	23/788	726/68,726	2,657/82,809	165,192/8,883,870
		myositis	1/788	5/68,726	89/82,809	3,016/8,883,870
		blood creatine phosphokinase increased	5/788	123/68,726	436/82,809	10,154/8,883,870
		musculoskeletal discomfort	7/788	214/68,726	764/82,809	44,608/8,883,870
	rosuvastatin	muscle spasms	7/1,022	326/68,726	1,669/77,853	78,652/8,883,870
		myalgia	10/1,022	264/68,726	2,725/77,853	68,379/8,883,870
		muscular weakness	12/1,022	281/68,726	1,070/77,853	44,118/8,883,870
		musculoskeletal pain	1/1,022	119/68,726	588/77,853	24,744/8,883,870
		rhabdomyolysis	6/1,022	62/68,726	1,130/77,853	13,921/8,883,870
		myopathy	10/1,022	726/68,726	3,097/77,853	165,192/8,883,870
		myositis	1/1,022	5/68,726	104/77,853	3,016/8,883,870
		blood creatine phosphokinase increased	1/1,022	123/68,726	42/77,853	10,154/8,883,870
		musculoskeletal discomfort	3/1,022	214/68,726	1,310/77,853	44,608/8,883,870
	simvastatin	muscle spasms	6/854	326/68,726	1731/102,851	78,652/8,883,870
		myalgia	5/854	264/68,726	2,755/102,851	68,379/8,883,870
		muscular weakness	6/854	281/68,726	1,290/102,851	44,118/8,883,870
		musculoskeletal pain	13/854	119/68,726	590/102,851	24,744/8,883,870
		rhabdomyolysis	5/854	62/68,726	1,296/102,851	13,921/8,883,870
		myopathy	23/854	726/68,726	2,678/102,851	165,192/8,883,870
		myositis	1/854	5/68,726	120/102,851	3,016/8,883,870
		blood creatine phosphokinase increased	2/854	123/68,726	40/102,851	10,154/8,883,870
		musculoskeletal discomfort	4/854	214/68,726	1,114/102,851	44,608/8,883,870

### 3.2 Signal preliminary screening

The relative risk and ratio of combined drug use were obtained as described previously nine. In the additive model, RDAB > RDA + RDB indicates that a suspicious ADR signal was generated. In the multiplicative model, RRAB/(RRA × RRB) > 1 indicates that a suspicious ADR signal was generated. After preliminary screening, five kinds of adverse reactions were identified for the combination of sacubitril/valsartan and atorvastatin. Both models indicated positive signals for rhabdomyolysis (difference 0.0004, ratio 1.6594), myopathy (difference 0.0051, ratio 1.6012), myositis (difference 0.0005, ratio 5.5099), and musculoskeletal discomfort (difference 0.0016, ratio 1.5527). The “blood creatine phosphokinase increased” (difference 0.0004, ratio 0.7696) showed a positive signal only in the additive model. For the combination of sacubitril/valsartan and rosuvastatin, both models indicated positive signals for muscle weakness (difference 0.0024, ratio 1.3458), myositis (difference 0.0002, ratio 4.4330), and “blood creatine phosphokinase increased” (difference 0.0001, ratio 1.5023). For the combination of sacubitril/valsartan and simvastatin, both models indicated positive signals for musculoskeletal pain (difference 0.0105, ratio 4.2686), myopathy (difference 0.0089, ratio 1.8207), myositis (difference 0.0003, ratio 4.6833), and “blood creatine phosphokinase increased” (difference 0.0013, ratio 3.8457). Notably, myositis and “blood creatine phosphokinase increased” showed positive results in the preliminary screening for all three combinations. The detailed results are presented in Table 2.

**TABLE 2:** Signal detection results of additive and multiplicative models.

Drug A	Drug B	Adverse reaction	Additive model			Multiplicative model		
			RD_AB_	RD_A_ + RD_B_	Difference	RR_AB_	RR_A_*RR_B_	Ratio
sacubitril/valsartan	atorvastatin	muscle spasms	0.0013	0.0077	−0.0064	1.1467	1.2533	0.9149
		myalgia	−0.0001	0.0188	−0.0189	0.9892	1.9685	0.5025
		muscular weakness	0.0039	0.0089	−0.0049	1.7888	2.4365	0.7342
		musculoskeletal pain	0.0010	0.0029	−0.0018	1.3669	1.4932	0.9154
		rhabdomyolysis	0.0048	0.0044	0.0004	4.0493	2.4402	1.6594
		myopathy	0.0106	0.0055	0.0051	1.5697	0.9803	1.6012
		myositis	0.0009	0.0005	0.0005	3.7380	0.6784	5.5099
		blood creatine phosphokinase increased	0.0052	0.0048	0.0004	5.5515	7.2131	0.7696
		musculoskeletal discomfort	0.0039	0.0023	0.0016	1.7691	1.1394	1.5527
	rosuvastatin	muscle spasms	0.0000	0.0085	−0.0084	1.0034	1.2974	0.7734
		myalgia	0.0050	0.0234	−0.0185	1.6487	2.2695	0.7265
		muscular weakness	0.0103	0.0079	0.0024	3.0665	2.2786	1.3458
		musculoskeletal pain	−0.0015	0.0037	−0.0052	0.4556	1.6857	0.2703
		rhabdomyolysis	0.0060	0.0123	−0.0062	4.8591	5.3326	0.9112
		myopathy	−0.0059	0.0132	−0.0191	0.6825	1.2154	0.5615
		myositis	0.0009	0.0007	0.0002	3.7380	0.8432	4.4330
		blood creatine phosphokinase increased	0.0001	0.0000	0.0001	1.1103	0.7391	1.5023
		musculoskeletal discomfort	−0.0012	0.0099	−0.0111	0.7582	2.0781	0.3649
	simvastatin	muscle spasms	−0.0018	0.0039	−0.0057	0.7936	1.0185	0.7791
		myalgia	−0.0018	0.0152	−0.0171	0.7607	1.7368	0.4380
		muscular weakness	0.0021	0.0067	−0.0046	1.4148	2.0794	0.6804
		musculoskeletal pain	0.0124	0.0019	0.0105	5.4653	1.2804	4.2686
		rhabdomyolysis	0.0043	0.0104	−0.0061	3.7363	4.6295	0.8071
		myopathy	0.0083	−0.0006	0.0089	1.4484	0.7955	1.8207
		myositis	0.0008	0.0006	0.0003	3.4492	0.7365	4.6833
		blood creatine phosphokinase increased	0.0012	−0.0001	0.0013	2.0490	0.5328	3.8457
		musculoskeletal discomfort	−0.0003	0.0039	−0.0042	0.9328	1.3377	0.6973

### 3.3 Statistical test of ADR signals

For a given drug combination, the proportion of adverse events observed approximately follows a binomial distribution (Hughes et al., 2023). Therefore, the SAS program “procgenmod” (with identity-link function for the additive model and log-link function for the multiplicative model) was used to analyze the preliminarily screened suspicious DDI signals. The positive results in Table 2 were statistically tested. The initial screening showed that the positive rate of the additive model was higher than that of the multiplicative model. However, after statistical testing, many signals were excluded as false positives. Among these, only the combination of “sacubitril/valsartan–simvastatin–musculoskeletal pain” had statistically significant associations in both models (*P* < 0.05). In contrast, “sacubitril/valsartan–atorvastatin–myopathy” and “sacubitril/valsartan–simvastatin–myopathy” showed significant associations only in the multiplicative model (*P* < 0.05). This suggests that the combination of sacubitril/valsartan with atorvastatin may increase safety risk to myopathy, while its combination with simvastatin may increase safety risks to musculoskeletal pain and myopathy. These combinations should be closely monitored in clinical practice. Results are shown in Table 3.

**TABLE 3:** Statistical tests of additive and multiplicative models.

Drug A	Drug B	Adverse reaction	Additive model (α = 0.05)		Multiplicative model (α = 0.05)	
			δ	*P*	exp(δ)	*P*
sacubitril/valsartan	atorvastatin	rhabdomyolysis	0.0004	0.8903	1.7047	0.2533
		myopathy	0.0054	0.3700	1.6449	0.0193
		myositis	0.0005	0.7163	5.6604	0.1153
		blood creatine phosphokinase increased	0.0005	0.8657	0.7906	0.6087
		musculoskeletal discomfort	0.0016	0.6245	1.5950	0.2263
	rosuvastatin	muscular weakness	−0.001	0.7623	1.0625	0.8381
		myositis	−0.0001	0.9282	3.4998	0.2548
		blood creatine phosphokinase increased	−0.0002	0.8674	1.1860	0.8667
	simvastatin	musculoskeletal pain	0.0106	0.0118	4.3445	< 0.0001
		myopathy	0.0092	0.0995	1.8660	0.0034
		myositis	0.0003	0.8259	4.0056	0.2005
		blood creatine phosphokinase increased	0.0013	0.4218	3.9149	0.0616

## 4 Discussion

Investigations of DDI are limited to pre-market clinical trials of pharmacokinetics, and it is impossible to evaluate all possible interactions of a new drug comprehensively (Zhao et al., 2023). Therefore, many DDI signals are not discovered until the drug is put on the market. More than one-third of elderly people in the United States take multiple medications, and 15% of these patients are at risk of severe DDIs (Qato et al., 2016). Patient exposure to DDIs can lead to significant reductions in health-related quality of life, significant prolongation of hospital lengths of stay, and even death (Hughes et al., 2023). Exposure of children in outpatient clinics to DDI can cause 14.6% of children to have increased blood drug concentrations, 13.6% of children to have neurological depression, and 9.9% of children to have prolonged QT interval (Kyler et al., 2024). While combined medication brings many curative effects to patients, it can also bring risks. Therefore, scientific monitoring of ADRs caused by DDI has important clinical significance. Sacubitril/valsartan is a relatively new drug, there are likely many ADRs and DDI that have not yet been discovered. It is usually used in combination with statins, but at present there are few studies on the safety of this drug combination. This study conducted risk signal mining in a publicly available dataset to evaluate safety of sacubitril/valsartan combined with atorvastatin, simvastatin, and rosuvastatin.

Considering ADRs of statins, those affecting the muscular system are the most recognizable and dangerous. They can be classified by severity as myalgia, myositis, and rhabdomyolysis, and are influenced by drug interactions, dose, and individual/genetic factors of the patient (Coste et al., 2019; Tournadre, 2020). Statins act as substrates for transporters of peptides OATP1B1 and OATP1B3, and therefore may interact with sacubitril/valsartan, which inhibit OATP1B1 and OATP1B3. Ayalasomayajula et al. (2016) conducted a study on the interaction of simvastatin and sacubitril/valsartan in 26 healthy people, and showed that sacubitril had an inhibitory effect on OATP1B1 and OATP1B3 *in vitro*. However, this effect did not cause changes in the pharmacokinetics of simvastatin. Ayalasomayajula et al. (2017) studied the drug interaction between atorvastatin and sacubitril/valsartan in healthy Chinese men. Their results showed that the maximum concentration of atorvastatin and its metabolites doubled. The area under the curve increased 1.3 times, but the adverse events did not increase with the increase in atorvastatin maximum concentration. However, the duration of the above studies was short, and the subjects were healthy people, which cannot fully reflect the ADRs caused by long-term increases in drug concentration during real-world clinical treatment. Patients with coronary heart disease commonly need to take statins for a long time, often combined with other underlying diseases (Salim S et al., 2023).

The results of the present study demonstrated that after preliminary screening and statistical testing, “sacubitril/valsartan–simvastatin–musculoskeletal pain” showed significant correlations in both models. In addition, “sacubitril/valsartan–atorvastatin–myopathy” and “sacubitril/valsartan–simvastatin–myopathy” were significantly correlated in the multiplicative model. This suggests that the combination of sacubitril/valsartan with atorvastatin may increase the risk of myopathy, while its combination with simvastatin may increase the risk of both musculoskeletal pain and myopathy. However, no signal was detected for any muscle system ADRs when sacubitril/valsartan was used in combination with rosuvastatin. This may be related to the smaller impact of inhibition of OATP1B1 and OATP1B3 on the AUC of rosuvastatin (Elsby et al., 2012). In the existing literature are five case reports of rhabdomyolysis caused by the combined use of sacubitril/valsartan and statins, four of which were associated with atorvastatin (Faber et al., 2016; Chan KH et al., 2020; Siew et al., 2022; Zhao FK et al., 2022) and one with rosuvastatin (Previsdomini et al., 2019). However, the present study did not detect a statistically significant association signal with rhabdomyolysis, consistent with findings from studies like (Sunaga and Ryo 2021; Tomiko and Yonezawa, 2021). This may be due to the relatively short time sacubitril/valsartan has been on the market, but it still warrants attention in future clinical use. Therefore, when atorvastatin and simvastatin are used in combination with sacubitril/valsartan in clinical treatment, close attention should be paid to adverse reactions in the muscular system, especially in patients with risk factors such as advanced age, abnormal liver and kidney function, and complex underlying diseases (Abraldes et al., 2016; Nisa et al., 2021). In addition, relevant adverse reaction reports should be continuously collected to determine whether there is a causal relationship between combined medication and adverse reactions.

Ideally, drug safety data are obtained from case-control and cohort studies, but these studies are often limited by factors such as patient variability, research methods, and ethical constraints. In contrast, this study evaluated adverse drug reactions (ADR) using data mining on a large dataset, which better reflects “real-world” drug safety and offers a valuable approach for assessing drug safety. The additive model has higher initial screening sensitivity than the multiplicative model and can identify more suspicious signals. The multiplicative model helps to enhance the credibility of the additive model’s signal detection. However, its sensitivity is limited by the number of event reports ratio calculations and low signal detection rate. Simultaneous use of the additive and multiplicative models can quickly and effectively detect DDI signals, which is beneficial to improving clinical drug use (YN, 2021). This study used additive and multiplicative models to successfully detect DDI signals of sacubitril/valsartan and statins, providing a reference for the safe clinical application of combined medication.

Furthermore, we observed a FAERS study conducted by (Sunaga and Ryo 2021; Tomiko and Yonezawa, 2021), which investigated the safety of rhabdomyolysis associated with the combination of sacubitril/valsartan and statins, and the commonly used PRR method was employed for signal detection, but the PRR method is primarily intended for single drug ADR signal detection. The criteria for statistical signal index ratio differ between solo drug use and combined use, leading to potential calculation discrepancies and a higher false positive rate (Susuta and Takahashi, 2014). Moreover, the study only tested for rhabdomyolysis. In contrast, our study utilized additive and multiplicative models for statistical analysis, mining adverse reaction signals across the entire muscular system. This approach resulted in a relatively robust and comprehensive ADR signal assessment of the muscular system. However, this study has some limitations. First, analyses were dependent on the data available in the database, and may not reflect biological correlations. Second, since sacubitril/valsartan has been on the market for a relatively short time, there is little data in the FAERS database on the adverse reactions related to its combination with statins, which may lead to biased results. Thirdly, due to the lack of multiple adverse reaction data for pravastatin, pitavastatin, and fluvastatin, these statins were not included in the present analysis. Finally, while the FAERS system has been utilized for post-marketing drug safety monitoring for decades and serves as a vast database of adverse drug reactions, this study solely utilized data from the FAERS database. Data from other sources such as the UK National Adverse Drug Reactions Database (Yellow Card Scheme), Australian Adverse Drug Reactions Database, and the Canadian Adverse Drug Reactions Database (Canada Vigilance) were not included in the statistical analysis. In the future, we aim to conduct integrated data mining across multiple databases and collaborate with clinical observational studies to more precisely evaluate the potential risks and benefits associated with combined drug usage in clinical practice.

## 5 Conclusion

Additive and multiplicative models were used to mine the risk signals of positive drug interactions between sacubitril/valsartan, atorvastatin, and simvastatin. Compared with a single drug, coadministration of sacubitril/valsartan with atorvastatin may increase safety risks to myopathy, with simvastatin may increase safety risks to the musculoskeletal pain and myopathy, which should be closely monitored in clinical practice. It is imperative to conduct long-term safety monitoring of the combination of sacubitril/valsartan and statins and to undertake related work in conjunction with clinical observational studies in the future. This approach will enable a more precise evaluation of the potential risks and benefits associated with actual clinical combinations.

## Data Availability

The original contributions presented in the study are included in the article/Supplementary Material, further inquiries can be directed to the corresponding author.

## Abbreviations

ADR, Adverse drug reactions; AUC, Area under the curve; DDI, Drug-drug interactions; FAERS, Food and Drug Administration Adverse Event Reporting System; OATP1B1, Organic anion transporting polypeptides 1B1; OATP1B3, Organic anion transporting polypeptides 1B3.
